# Microbial pathogens induce neurodegeneration in Alzheimer’s disease mice: protection by microglial regulation

**DOI:** 10.1186/s12974-021-02369-8

**Published:** 2022-01-06

**Authors:** Tal Ganz, Nina Fainstein, Amit Elad, Marva Lachish, Smadar Goldfarb, Ofira Einstein, Tamir Ben-Hur

**Affiliations:** 1 Faculty of Medicine, Hebrew University of Jerusalem, Jerusalem, Israel; 2 The Department of Neurology, The Agnes Ginges Center for Human Neurogenetics, Hadassah-Hebrew University Medical Center, Jerusalem, Israel; 3 Department of Physical Therapy, Faculty of Health Sciences, Ariel University, Ariel, Israel

**Keywords:** Alzheimer's disease, Infection, Pathogen associated molecular patterns, Lipopolysaccharides, Neurodegeneration, Neuroinflammation, Microglia, Retinoic acid receptor

## Abstract

**Background:**

Neurodegeneration is considered the consequence of misfolded proteins’ deposition. Little is known about external environmental effects on the neurodegenerative process. Infectious agent-derived pathogen-associated molecular patterns (PAMPs) activate microglia, key players in neurodegenerative diseases. We hypothesized that systemic microbial pathogens may accelerate neurodegeneration in Alzheimer’s disease (AD) and that microglia play a central role in this process.

**Methods:**

We examined the effect of an infectious environment and of microbial Toll-like receptor (TLR) agonists on cortical neuronal loss and on microglial phenotype in wild type versus 5xFAD transgenic mice, carrying mutated genes associated with familial AD.

**Results:**

We examined the effect of a naturally bred environment on the neurodegenerative process. Earlier and accelerated cortical neuron loss occurred in 5xFAD mice housed in a natural (“dirty”) environment than in a specific-pathogen-free (SPF) environment, without increasing the burden of Amyloid deposits and microgliosis. Neuronal loss occurred in a microglia-rich cortical region but not in microglia-poor CA regions of the hippocampus. Environmental exposure had no effect on cortical neuron density in wild-type mice. To model the neurodegenerative process caused by the natural infectious environment, we injected systemically the bacterial endotoxin lipopolysaccharide (LPS), a TLR4 agonist PAMP. LPS caused cortical neuronal death in 5xFAD, but not wt mice. We used the selective retinoic acid receptor α agonist Am580 to regulate microglial activation. In primary microglia isolated from 5xFAD mice, Am580 markedly attenuated TLR agonists-induced iNOS expression, without canceling their basic immune response. Intracerebroventricular delivery of Am580 in 5xFAD mice reduced significantly the fraction of (neurotoxic) iNOS + microglia and increased the fraction of (neuroprotective) TREM2 + microglia. Furthermore, intracerebroventricular delivery of Am580 prevented neurodegeneration induced by microbial TLR agonists.

**Conclusions:**

Exposure to systemic infections causes neurodegeneration in brain regions displaying amyloid pathology and high local microglia density. AD brains exhibit increased susceptibility to microbial PAMPs’ neurotoxicity, which accelerates neuronal death. Microglial modulation protects the brain from microbial TLR agonist PAMP-induced neurodegeneration.

## Background

The neurodegenerative process in Alzheimer’s disease (AD) is considered the consequence of deposition of misfolded amyloid-β (Aβ) and hyperphosphorylated tau (p-tau) proteins (1), with histopathological hallmarks that include Aβ-rich extracellular plaques, p-tau-rich neurofibrillary tangles, microgliosis, astrogliosis, and neuronal loss. Less is known on how the external and systemic environments affect the brain and disease. Accumulating evidence imply an association between systemic infections and AD. Systemic infections are associated with long-lasting cognitive decline in patients with pre-existing AD (2,3). However, it is not clear whether and how systemic infections affect the neurodegenerative process itself.

The bacterial wall contains pathogen-associated molecular patterns (PAMP), which activate the host immune system. A major group of PAMP is the lipopolysaccharide (LPS) bacterial endotoxins, which are a major component of the outer membrane of gram-negative bacteria. Soluble endotoxin is released when bacteria are destroyed but is also released physiologically as outer membrane vesicles (4). When released, endotoxin causes inflammatory activation mainly via activating TLR4 on the cell surface of innate immune cells, including microglia. Animal studies have shown that systemic bacterial endotoxins can induce brain inflammation with accompanying inflammatory-cytokine -induced sickness behavior and cognitive dysfunction (5–7). Furthermore, endotoxin has been shown to exacerbate brain pathology in animal models, and specifically Aβ production and aggregation (8) and Tau hyperphosphorylation (8,9). These findings are of clinical relevance, as studies found a threefold increase in mean blood endotoxin levels, a two- to threefold increase in brain endotoxin levels in AD patients, and up to a 26-fold increase in hippocampal tissue (10). Endotoxin is also found in amyloid plaques (11,12). Indeed, people with chronic gingival disease (periodontitis) have elevated blood endotoxin (13), a higher risk of AD (14), and a faster rate of cognitive decline (13,15,16).

Microglia are the principal innate immune cell type in the CNS, which sense potential pathogens and detect disruptions in tissue homeostasis by several receptor families, collectively referred to as pattern recognition receptors (PRRs) (17). Within the Toll-like receptor (TLR) family of PRR's, several are expressed by microglia cells and drive the neuroinflammatory response to PAMPs (18). TLR–ligand interactions trigger a series of signaling events resulting in the production of inflammatory factors, reactive oxygen species (ROS), and the release of acute-phase proteins. In addition, microglia are increasingly appreciated as key players in neurodegenerative diseases (19,20), activated to a neurotoxic state by misfolded Aβ and p-tau (18–23). Neurotoxic microglia contribute to the progression of AD by failing to clear Aβ plaques, and damage neurons via nitric oxide, ROS, and cytokines production. Microglial TLRs bind Aβ (24) and were recently suggested to mediate the neurotoxicity of microglia to brain neurons in AD (25,26). Since microbial endotoxins are powerful TLR4 agonists and stimulate microglia as well (27), we hypothesized that: (1) infectious agents and PAMP may accelerate neurodegeneration in the AD brain; (2) microglia may play a central role in this process; and (3) microglial modulation may protect the brain from PAMP-induced neurodegeneration.

Transgenic 5xFAD mice carry a cassette of five human familial AD genes under the Thy1.1 promoter, leading to marked AD pathology, including heavy Amyloid β deposition and marked astrogliosis and microgliosis. While these pathological changes are associated with memory impairment, 5xFAD mice exhibit only mild loss of cortical neurons (28,29). This provides a unique opportunity to study the effects of external insults on the neurodegenerative process in brains inflicted with AD pathology. We, therefore, examined whether systemic infections and bacterial endotoxin cause neurodegeneration in wild type (wt) and AD mice. We first found that 5xFAD (but not wt) mice that were housed in natural, non-SPF (so-called “dirty”) conditions exhibited early and accelerated loss of cortical neurons, as compared to mice that were housed in SPF conditions. Neuronal loss was observed in a cortical region which displayed high microglial density, but did not occur in the CA1 and CA3 regions of the hippocampus which displayed tenfold lower microglial density. To model the insult produced by systemic infections, we injected LPS systemically into 5xFAD mice. The AD pathology was associated with significantly increased susceptibility to LPS neurotoxicity. Modulation of microglial neurotoxicity using a retinoic acid receptor α (RAR-α) agonist, delivered to the brain, protected it from LPS-induced neurodegeneration. We suggest that systemic microbial infections may directly accelerate the neurodegenerative process in distinct brain regions inflicted with AD pathology.

## Methods

### Animals

A colony of 5xFAD mice was initially established by mating C57Bl/6J mice (originally supplied by Jackson) with heterozygote 5xFAD mice (5xFAD transgenic mouse model, carrying 5 mutated human genes associated with familial Alzheimer’s disease, APP K670N/M671L (Swedish), APP I716V (Florida), APP V717I (London), PS1 M146L, PS1 L286V), both a generous gift of Prof. Dani Frenkel from Tel Aviv University. Offspring of further generations were screened for carrying the transgenic cassettes (see below). To ascertain that 5xFAD and wild-type (wt) control mice had identical genetic backgrounds and environments, further breeding was performed by mating a heterozygote transgene-positive with a transgene-negative mouse, and all littermates were housed together. We made sure that each litter contained approximately 50% transgene-positive mice. Both male and female mice were used, maintaining equal distribution between experimental groups. Breeders were not used for experiments. Animal experimentation was approved by the institutional ethics committee, approval number MD-19-15966-5.

### Mice genetic screening

Mice tails were sampled for polymerase chain reaction (PCR) analysis of APP, PS1, and a control mouse gene. DNA was extracted from mice tails using 50 mM Tris HCl (pH = 8), 0.05% Triton X-100, and 19.6 mg/mL Proteinase K. The vials were then heated to 55 °C for 15 min followed by 5 min in 80 °C. The PCR reaction mixture included 5 μL of DNA, 300 nM of the appropriate forward and reverse primers (Syntezza, Israel), and 5 μL of the master mix buffer containing nucleotides and Red Load Taq polymerase (Larova) in a total volume of 25 μL. Gene amplification was carried out using the GeneAmp 9700 Sequence Detection System (Applied Biosystems). For APP analysis, amplification included one stage of 3 min at 94 °C, followed by 35 cycles of a 3-step loop: 30 s at 94 °C, 1 min at 55 °C, and 1 min at 72 °C, followed by 2 min in 72 °C and cooling to 10 °C. For PS1 analysis, 35 cycles of a 3-step loop, 20 s at 94 °C, 1 min at 60 °C, and 1 min at 72 °C, followed by 2 min in 72 °C and cooling to 10 °C.

### ICV injections

Mice were anesthetized using a combination of ketamine (80 mg/kg; i.p.) and xylazine (20 mg/kg; i.p.) prepared in normal saline. Single intracerebroventricular (ICV) injection of LPS or Zymosan (in either 10 μg or 25 μg) was performed using a stereotactic device, at coordinates *A* = 0, *L* = 1, *H* = 2.5. Mice were sacrificed the next day or 3 days following the ICV injection.

### Am580 solution preparation and surgical insertion of ICV pump

The selective RARα agonist, Am580 (Cayman chemicals, 15261), was dissolved in 8% DMSO, 2% Castor oil, and 90% saline, to a concentration of 28.5 mM. Mice were anesthetized as described above and an ICV Alzet pump (1007D or 1004) was surgically placed, allowing continuous slow-release delivery of Am580 for either 7 or 28 days. The pump cannulas were inserted into the lateral ventricle, as described above. Pump placement and fixation were performed according to the manufacturer’s instructions. Sham-operated mice were inserted with a pump containing the vehicle only.

### IP injections of LPS

Three repeated injections of LPS (LPS from *E. coli* O111:B4, L2630, Sigma-Aldrich, 150 μg/animal/injection) were performed on days 0, 3, and 5. Animals were sacrificed on day 7 by perfusion.

### Histopathology

Animals were anesthetized with a lethal dose of pentobarbital, and the brains were perfused via the ascending aorta with ice-cold phosphate-buffered saline followed by cold 4% paraformaldehyde. Tissues were deep-frozen on dry ice, serial 10 μm coronal sections were prepared, and immune-fluorescent stainings were performed as previously described (30). The following antibodies were used: mouse anti-NeuN (1:200, Chemicon), rabbit anti-Iba1 (1:220, Wako), anti-iNOS (1:1000, Novus biologicals), anti-β amyloid (1:800, BioLegend). Goat anti-rabbit Alexa-fluor 488 (1:200, Invitrogen), goat anti-rat Alexa-fluor 488 (1:200, Invitrogen), and goat anti-mouse Alexa-fluor 555 (1:200, Invitrogen) were used as secondary antibodies where appropriate. Nuclear counterstain was performed using DAPI (Vector Laboratories).

### Quantification of neuron density

Each mouse brain was evaluated in coronal sections at the level of Bregma 0.0 ± 0.1 mm for cortical assessment, and at Bregma − 2 ± 0.1 mm for hippocampal assessment. To measure neuronal cell density in the cortex, two adjacent × 20 magnification microscopic images of Dapi-stained nuclei/NeuN were obtained in a blinded manner from each cortical hemisphere, midway between the pial lining and the corpus callosum (4 sections, 4 fields per section for each brain). To measure neuronal cell density in the hippocampus, two × 20 magnification microscopic images of Dapi-stained nuclei were obtained in a blinded manner from each hemisphere (at CA1 and CA3 regions). Images were counted manually in a blinded manner adhering to stereological principles.

### Isolation and growth of adult brain microglia

Microglia isolation was performed on 7-month-old 5xFAD and wt mice. Mice were terminally anesthetized and decapitated, and the brains were excised into Earl’s based salt solution. The brains were dissociated to a single-cell suspension using the Neural Tissue Dissociation Kit (P) (Miltenyi Biotec, 130–092–628) according to the manufacturer’s protocol. For myelin removal, Percoll (GE Healthcare) protocol was performed. Microglia were isolated from myelin-free single-cell suspension using CD11b-conjugated beads (Miltenyi Biotec, 130–093-634) according to the manufacturer’s protocol followed by depletion with MS columns (Miltenyi Biotec, 130-042-201). The procedure resulted in > 90% Cd11b + cells and no additional gating was required for FACS experiments. The cells were counted, followed by either FACS staining or seeded for in vitro experiments.

Isolated microglia were seeded (60,000 cells/mL) in a six-well poly-l-lysin coated plate, in DMEM medium supplemented with 20% fetal bovine serum, 20% l-medium, l-glutamine, sodium pyruvate, and Pen-strep. l-medium is a collected conditioned medium of 1-week confluent cultured l-cells (a generous gift of Prof. Shlomo Rotshenker). Microglia reached confluency after 72 h. Confluent cells were pretreated for 12 h with 100uM Am580 dissolved in DMSO. DMSO percentage did not exceed 0.1% of the total medium. Some experimental groups were then treated with 12 h of either LPS stimulation (200 ng/mL, *E. coli*, 111:B4, Merck) or Zymosan (200 ng/mL, Saccharomyces cerevisiae, InvivoGen). RNA extraction was performed 12 h later.

### Latex beads phagocytosis

Microglia were isolated as described above and seeded (150,000 cells/mL) in a 24-well plate with glass coverslips coated with poly-l-lysin. Microglia were cultured with LPS for 1 h, followed by 30 min of exposure to latex beads (4 µl/1 mL, Sigma-Aldrich (, cell fixation, and Iba1 staining (1:220, Wako). Images were taken using Nikon confocal AR1 + microscope. Uptake analysis was quantified manually in a blinded manner.

### FACS analysis

Microglia were isolated from the brains as described above. TREM2 expression was assessed immediately after the isolation using Alexa-fluor 488-anti-human and mouse Trem2 (237920, R&D systems) by fluorescence-activated cell sorter (FACS) analysis (Beckman Coulter).

### Real-time PCR

RNA was isolated from microglia using RNeasy Plus Mini Kit (Qiagen). cDNA was generated from a concentration of 50 μg/mL RNA using qScript cDNA synthesis Kit (Quanta Biosciences) according to manufacturer’s instructions. The reaction mixture included 1 μL of cDNA, 300 nM of the appropriate forward and reverse primers (Agentek), and 5 μL PerfeCTA SYBR Green FastMix ROX (Quanta Biosciences) to a total volume of 10 μL. Gene amplification was carried out using the StepOnePlus real-time PCR system (Applied Biosystems). The following primers were used:

TNFa F: 5’ cagaccctcacactcagatcatctt; TNFa R: 5’ cctccacttggtggtttgctac.

iNOS F: 5’ cccagccttgcatcctcat; iNOS R: 5’ atgcggcctcctttgagc.

IL1 b F: 5’ cctgaactcaagtgtgaaatgcc; IL1 b R: 5’ tcatcaggacagcccaggtc.

IL10 F: 5′ cagccaggtgaagactttctttc; IL10 R: 5′ ctgcattaaggagtcggttagca.

### Endotoxin concentrations measurement

Endotoxin concentrations were determined with Pierce LAL Chromogenic Endotoxin Quantitation Kit (Thermo Fisher Scientific) following the manufacturer’s instructions. In this kit, the measurement of one endotoxin unit/mL equals approximately 0.1 ng endotoxin/mL.

### Statistical analysis

Statistical analysis was performed using Prism, version 8. The statistical tests used in this manuscript were students’ one-way unpaired *T* test or one-way analysis of variance (ANOVA) followed by Tukey's post-hoc test, as appropriate. The test used is specified for each experiment in the figure legends. *P* value was considered significant < 0.05. The number of repeats and the number of samples (*n*) in each experimental group are specified in figure legends.

## Results

### An infectious environment accelerates neurodegeneration in AD mice

We first examined the effect of a naturally bred (so-called “dirty”) environment, as compared to an SPF environment, on the neurodegenerative process in 5xFAD versus wt mice. Groups of 5xFAD and wt mice were housed in an SPF facility or transferred to a non-SPF animal facility at the age of 3 months and followed clinically. Importantly, there were no recorded infections with clinical manifestations or major signs of sickness behavior during the course of the experiment in either group. We examined brain pathology and density of cortical neurons at two time points: ages 7 months and 1 year. In 5xFAD mice, the transgenic cassette is expressed under the Thy1.1 promoter, and the AD pathology is disseminated throughout the cortex, rather than being localized to the Hippocampus, as occurs in the early stages of the classical amnestic form of human AD (31,32). Therefore, the pathological evaluation was performed both in the frontal cortex and hippocampus.

**Fig. 1:**
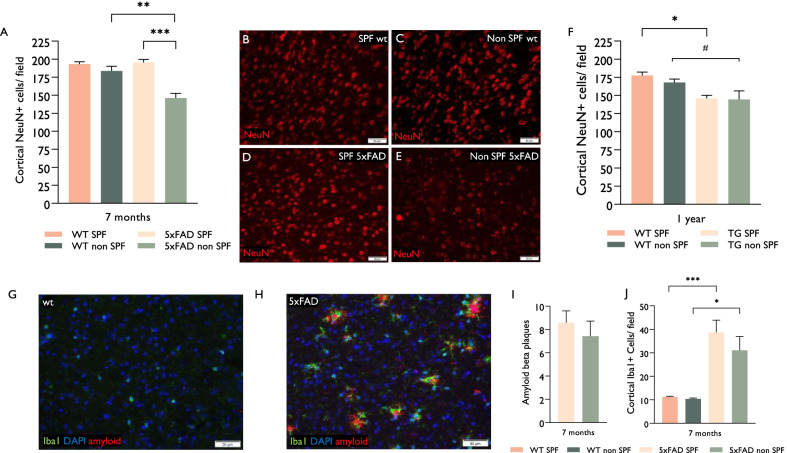
Infectious environment accelerates neurodegeneration in frontal cortex of AD mice. Wt and 5xFAD mice were grown in either naturally bred (so called “dirty”) environment or SPF environment. Mice were sacrificed at two timepoints for pathological assessment. **A** In wild type mice there was no loss of cortical neurons at age 7 months, whether grown in an SPF or in a naturally bred, non-SPF facility. 5xFAD mice grown in a non-SPF environment exhibited earlier and accelerated neurodegeneration as compared to 5xFAD mice grown in a SPF-facility, evident already at age 7 months. **B**–**E** Representative images of the cortex (at Bregma 0.0 ± 0.1 mm) stained for NeuN + neurons. **B** 7-month WT mice in an SPF environment. **C** 7-month WT mice in a non-SPF environment. **D** 7-month 5xFAD transgenic mice in a SPF environment. **E** 7-month 5xFAD transgenic mice in a non-SPF environment. **F** At age 1 year, there was a reduction of cortical neuron density in both 5xFAD groups compared to wt, with no further contribution of environmental conditions. **G**–**H** Representative images of the cortex stained for Iba1 + microglia and amyloid-beta plaques in wt and 5xFAD transgenic mice age 7 months. **I** Amyloid plaques were found only in transgenic mice, with no significant difference between SPF and non-SPF groups. **J** 5xFAD mice exhibited three- to fourfold increase in microglial density as compared with Wt mice and no difference between those housed in non-SPF versus SPF conditions. Error bars-SEM. *P* values by students unpaired *T* test in I and one-way analysis of variance (ANOVA) followed by Tukey’s post-hoc test in **A**, **F**, **I**: #*P* = 0.06, *P < 0.05, **P < 0.01, ***P < 0.001. *n* = 5–6, excluding 7-month non-SPF 5xFAD in which *n* = 4

In wild-type mice, there was no difference in cortical neurons' density at age 7 months, between those housed in an SPF facility or a non-SPF facility (Fig. 1A). In 5xFAD mice housed in an SPF facility, the counts of cortical neurons at age 7 months were similar to wt mice, indicating a lack of neurodegeneration, as previously described (29,33). 5xFAD mice housed in a natural environment exhibited earlier neurodegeneration in the frontal cortex as compared to 5xFAD mice grown in an SPF facility: at the age of 7 months, these mice had 25.3 ± 3.38% fewer cortical neurons per field, as compared to their SPF-housed counterpart (Fig. 1A–E). At age 1 year, there was a borderline significant reduction of cortical neuron density in both 5xFAD groups compared to wt, with no further contribution of environmental conditions (Fig. 1F).

We compared whether the increase in neurodegeneration in 5xFAD mice housed in a natural environment was associated with enhanced AD pathology. To that end, Amyloid β deposition and microgliosis were evaluated in the cortex of 7-month-old mice (Fig. 1G–J). 5xFAD mice exhibited Aβ-rich plaques (Fig. 1G–I) and a three- to fourfold increase in the density of microglia as compared to wt mice (Fig. 1G–J), indicating a heavy burden of AD pathology, as previously described (29). However, there was no difference in the density of Amyloid plaques (Fig. 1I) or in the number of microglia (Fig. 1J) in the cortex of 5xFAD mice housed in a natural environment, as compared to 5xFAD mice housed in an SPF facility.

**Fig. 2:**
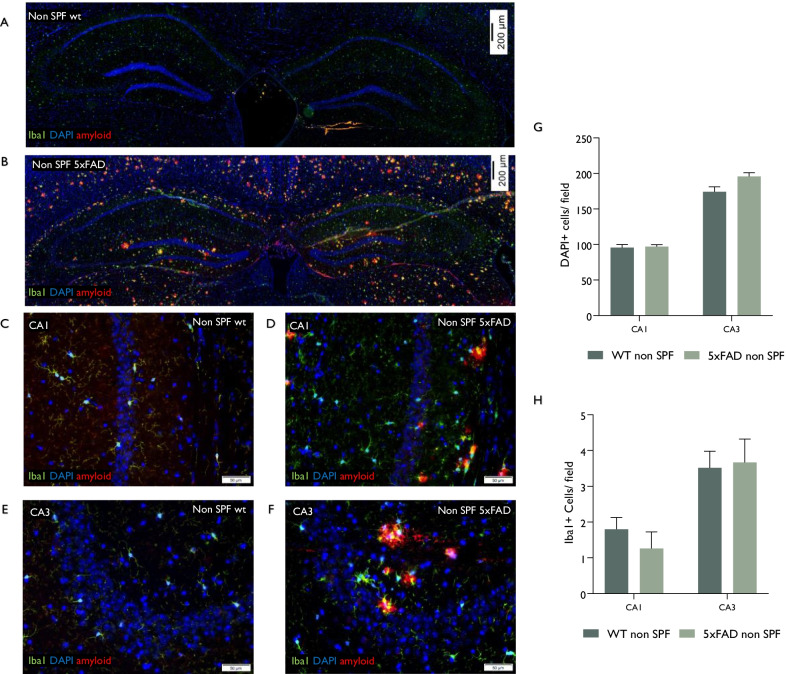
Infectious environment does not accelerate neurodegeneration in microglia-poor regions of the hippocampus. Wt and 5xFAD mice were housed in a naturally bred environment. Mice were sacrificed at age 7 months for pathological assessment. **A**–**F** Representative images of coronal brain sections at Bregma -2 ± 0.1 mm stained for DAPI, Iba1, and amyloid-beta. hippocampus (**A**, **B** total hippocampus; **C**, **D** CA1; **E**, **F** CA3). There was no significant difference in hippocampal neuronal density between 5xFAD and wt mice (**H**). The density of CA1 and CA3 Iba1 + microglia was significantly lower than cortical microglia (Fig. 1J), and there was no difference between 5xFAD and wt mice (**G**). Error bars-SEM. *P* values by one-way analysis of variance (ANOVA) followed by Tukey’s post-hoc test, *n* = 6/group

To examine whether environmental exposure of the non-SPF conditions is associated with neuronal loss in other brain regions as well, and is related to local microglial density, we quantified neuron density in the CA1 and CA3 regions of the hippocampus in 5xFAD vs. wt mice, housed in the non-SPF conditions (Fig. 2A–G). Both CA1 and CA3 regions displayed similar neuronal counts between wt and 5xFAD mice (Fig. 2G). Microglial density in the neuron-packed CA1 and CA3 regions was approximately tenfold lower than in the frontal cortex (Figs. 1, 2), and there was no difference in their density between wt and 5xFAD mice housed in a natural environment (Fig. 2A–G).

Thus, a naturally bred, non-SPF environment is associated with accelerated neurodegeneration in AD mice, without an increase in AD pathology. The accelerated neurodegeneration occurred in cortical areas with high but not low microglial density. This raises the possibility of direct neurotoxicity by microbial elements, such as pathogen-associated molecular patterns (PAMP), to which the AD brain is exposed in the natural bred environment, and may be mediated by neurotoxic microglia.

### Modeling infection-induced neurodegeneration by delivery of microbial PAMPs in AD mice

The finding of accelerated neurodegeneration in AD mice that were housed in (infectious) natural conditions, suggested that PAMP produced by infectious agents that colonize mice in the non-SPF environment may induce neurodegeneration in the brains of mice, inflicted with AD pathology. The SPF facility precludes the infection/colonization of mice with multiple microorganisms (Table 1). These include several gram-positive bacteria, which comprise various glycolipids that act as TLR2 agonists, and several gram-negative bacteria, harboring various endotoxins (lipopolysaccharides, LPS), that act on TLR4 (Table 1). The lack of change in AD pathology suggests a direct effect of pathogen-derived factors on the loss of cortical neurons.

**Table 1:** Specific pathogen free environment

Bacteria, Mycoplasma and Fungi	Gram stain	Additional information
Pasteurella pneumotropica	Gram negative	Oropharynx, opportunistic, LPS +
Pseudomonas aeruginosa	Gram negative	Opportunistic, LPS +
Klebsiella pneumonia	Gram negative	Opportunistic, LPS +
Klebsiella oxytoca	Gram negative	Opportunistic, LPS +
Pasteurellacaea	Gram negative	LPS +
Citrobacter rodentium	Gram negative	Colon, LPS +
Salmonella spp.	Gram negative	LPS +
Streptobacillus moniliformis	Gram negative	Nasopharynx, LPS +
CAR bacillus	Gram negative	Respiratory tract
Hellicobacter spp.	Gram negative	Upper GI, LPS +
Streptococcus pneumoniae	Gram positive	Respiratory tract, opportunistic, LTA +
Streptococcus B-haemolytic	Gram positive	Skin, LTA +
Staphylococcus aureus	Gram positive	Skin, opportunistic, LTA +
Corynebacterium bovis	Gram positive	
Corynebacterium kutscheri	Gram positive	
Clostridium piriforme (Tyzzer’s disease)	Gram positive	
Mycoplasma pulmonis	No bacterial wall	Respiratory tract
Pneumocystis murina		Fungus
Viruses and parasites		

The SPF facility excludes various infections, from pathogens varying from viruses, to bacteria and parasites, by routinely examining the animals. The SPF environment exclude bacterial pathogens that may colonize and infect (some of them opportunistic infections) the oral and nasopharyngeal mucosa, the respiratory tract the upper and lower GI tract. 10 out of 17 strains are gram negative bacteria which produce LPS

Mouse hepatitis virus (MHV), Mouse rotavirus (EDIM), Mouse Parvo virus, Minute virus of mice (MVM), Theiler’s murine encephalomyelitis virus (GD-7), Mouse Norovirus (MNV), Pneumonia virus of mice, Sendai virus (SEND), Ectromelia virus (ECTR), Reovirus type 3 (REO-3), Lymphocytic choriomeningitis virus (LCMV), Mouse Adenovirus type 1 (MAD-1), Mouse Adenovirus type 2 (MAD-2), Mouse cytomegalovirus, Mouse Polyoma virus, Ectoparasites, Intestinal helminths, Enteric protozoa

**Fig. 3:**
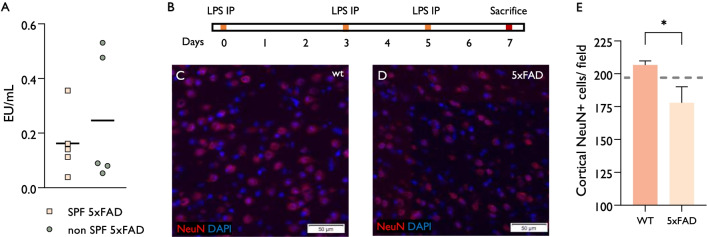
Modelling infection-induced neurodegeneration by delivery of microbial PAMPs in AD mice. **A** Serum endotoxin levels were measured in 5xFAD mice from either SPF or naturally bred environments at age 7 months. Among the naturally bred environment group, more mice had high serum endotoxin levels, but the difference between the two groups was not significant. **B** LPS was administered by three intraperitoneally injections to either wt or 5xFAD mice, grown in an SPF environment. **C**, **D** Representative images of the cortex stained for NeuN + neurons. **C** Wt mice, **D** 5xFAD mice. **E** Systemic LPS injection did not affect cortical neuronal density in wt mice (*n* = 4) as compared to the standard density in 7-month-old mice (represented by dashed line) but caused a significant decrease in cortical neuronal density in 5xFAD mice (*n* = 5). Error bars-SEM. *P* values by students unpaired *T* test: *P < 0.05

We measured the serum endotoxin level in transgenic mice from either SPF or naturally bred environments prior to sacrifice at age 7 months (Fig. 3A). Among the naturally bred environment group, more mice had high serum endotoxin levels, but the difference between the two groups was not significant. We presume that incidental systemic sub-clinical infections in the natural environment cause transient elevations in endotoxin levels, and, therefore, not detected in all mice by a single random sampling.

We hypothesized that recurrent sub-clinical endotoxemias may cause neurodegeneration in mice housed in a natural environment. To examine the effect of systemic endotoxins, we administered LPS to 7-month-old SPF-housed 5xFAD and wt mice followed by an examination of cortical neuronal density in the frontal lobe region, which displayed accelerated neurodegeneration in the natural environment. LPS was administered by three intraperitoneal injections, at a sufficiently low dose, not to cause a clinical effect, to resemble sub-clinical infections. Systemic LPS injections did not affect cortical neuronal density in wt mice. In comparison, LPS injections caused a significant 14 ± 11% decrease in cortical neuronal density in 5xFAD mice (Fig. 3B–E). These findings indicate increased vulnerability of the 5xFAD brain, inflicted with AD pathology to the neurotoxic effect of LPS.

Thus, delivery of PAMP to AD mice can model the neurodegenerative process caused by the infectious environment and provides a useful platform to examine the protective effects of therapeutic agents against (TLR agonist-induced) neurodegeneration.

### Modulation of brain microglia prevents PAMP-induced neurodegeneration

Since brain TLR4 is expressed mostly by microglia, we hypothesized that microglia mediate LPS-induced neuronal death and that modulation of microglia may reduce endotoxin-induced microglial neurotoxicity. As complete silencing of microglia by removal or by locking them in a non-responsive state is detrimental to the brain (34), it is advisable to use a compound that reduces microglial activation without paralyzing them. Previous studies suggested that retinoids can suppress macrophage and microglial activation (35,36), and yet enhance some of their physiologic functions, such as supporting phagocytosis and remyelination (37–39). We, therefore, used here the selective retinoic acid receptor alpha (RARα) agonist Am580, which modulates microglial activation, and specifically regulates microglial response to LPS (40).

**Fig. 4:**
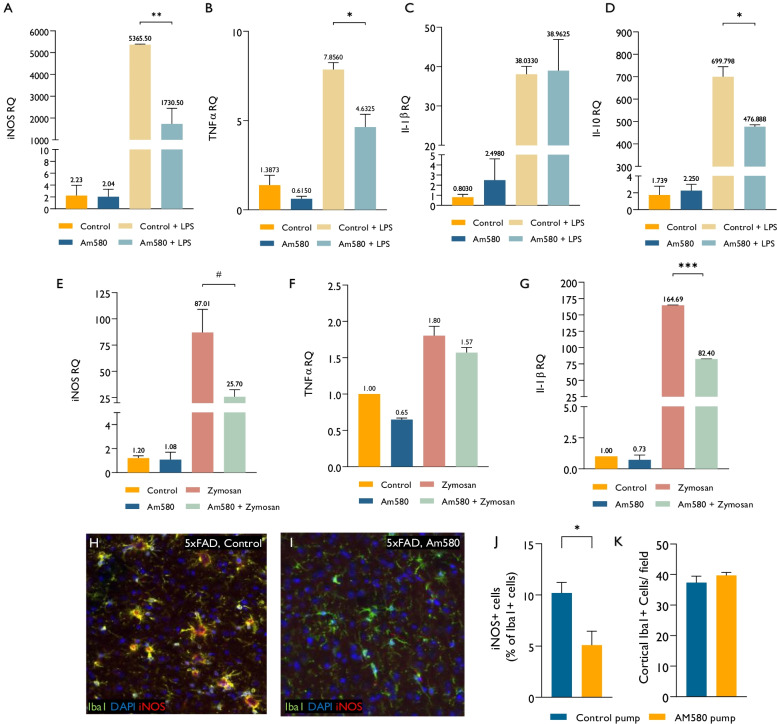
Am580 reduces microglial toxicity. The effect of Am580 on naïve or LPS-stimulated CD11b + microglia isolated from 7-month-5xFAD transgenic mice was examined. **A**–**D** Data from a representative experiment. Am580 significantly inhibited the expression levels of iNOS (**A**) and TNFα (**B**) RNA in microglia stimulated by LPS. Am580 had no significant effect on the expression of Il-1b (**C**) and had a suppressor effect on Il-10 expression (**D**). Activation of microglia with Zymosan caused increase in iNOS, TNFα and Il-1b expression, which were effectively attenuated by pre-exposure to Am580 (**E**–**G**). Am580 was given continuously in an ICV delivery, using mini-osmotic pumps for 4 weeks in 7-month-old 5xFAD mice (*n* = 4). **H**, **I** Representative images of the cortex stained for Iba1 + microglia. Am580 treated mice had significant decrease in the fraction on iNOS + toxic microglia without affecting the total number of Iba1 + cells (**J**–**K**, *n* = 4). Error bars-SD. *P* values by students unpaired T test in J and one-way analysis of variance (ANOVA) followed by Tukey’s post-hoc test in **A–D** #*P* = 0.06, *P < 0.05, **P < 0.01. **E**–**G** show representative data.

First, primary microglia were isolated from 7-month-old 5xFAD mice. Following 3 days in culture, the cells were treated with Am580 for 12 h and then activated with LPS for another 12 h. Am580 treatment caused marked inhibition of LPS-induced iNOS expression (Fig. 4A). Am580 mildly inhibited LPS-induced TNFα expression (Fig. 4B), had no significant effect on Il-1b expression (Fig. 4C), and significantly reduced the LPS-induced expression of Il-10 (Fig. 4D). To confirm that the immune-modulatory effect of Am580 was not restricted to TLR4 activation, we examined whether Am580 affects also microglial activation in response to a TLR2 agonist. Zymosan is a powerful β-Glucan polysaccharide TLR2 agonist derived from the yeast Saccharomyces cerevisiae, that induces microglial activation and neurodegeneration in 5xFAD mice (33). Activation of microglia with Zymosan caused a marked increase in iNOS expression, which was effectively attenuated by pre-exposure to Am580 (Fig. 4E). Am580 attenuated Zymosan-induced IL1b expression, but not TNFα expression (Fig. 4F–G). Thus, in agreement with other observations (41), Am580 may reduce the neurotoxic phenotype of microglia, induced by TLR agonists, such as LPS and Zymosan, with a milder effect on their basic immune function.

Next, we evaluated in vivo effects of Am580 following its continuous intra-cerebro-ventricular (ICV) delivery, using mini-osmotic pumps for 4 weeks in 7-month-old 5xFAD mice. 5xFAD mice exhibited a large fraction of iNOS + microglia (Fig. 4H). In Am580-treated 5xFAD mice, there was a significant decrease in the fraction of iNOS + microglia (as compared to vehicle-treated 5xFAD mice) (Fig. 4I, J), without affecting the total number of Iba1 + cells (Fig. 4K).

**Fig. 5:**
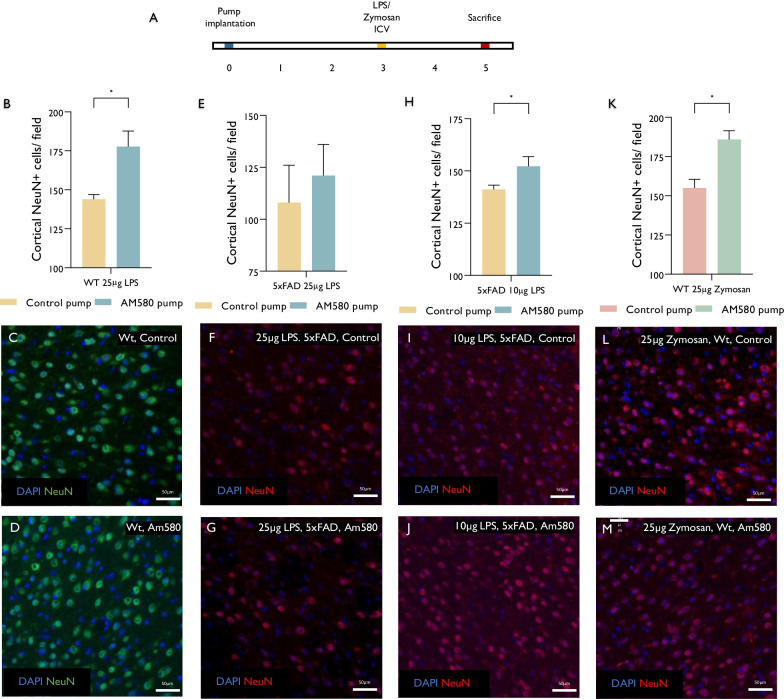
Modulation of brain microglia prevents PAMP-induced neurodegeneration. A single 25 µg LPS dose was injected ICV into 7-month-old wt mice, in which Am580 (or vehicle) was delivered continuously via mini-osmotic pumps and mice were sacrificed after 2 days for quantifying cortical neuronal density (**A**). ICV injection of LPS caused a significant loss in cortical neurons in wt mice, which was partly prevented by Am580 (**B**–**D**, *n* = 4). ICV injection of a similar dose of LPS into 5xFAD mice caused profound neuronal loss, which was not affected by Am580 (**E**–**G**, *n* = 5). Injection of 10 µg LPS caused moderate loss of cortical neurons, which was partly prevented by Am580 treatment (H-J, wt, *n* = 4, 5xFAD, *n* = 5). To confirm that the neuroprotective effect of the RARα agonist was not restricted to TLR4-mediated neurotoxicity of LPS, we injected 25 µg Zymosan ICV to wt mice in which Am580 (or vehicle) was delivered continuously via mini-osmotic pumps. Am580 provided efficient protection against Zymosan-induced cortical neuronal loss (**K**–**M**, wt, *n* = 7 5xFAD, *n* = 5), supporting the notion that microglial modulation may protect from PAMP-induced neurodegeneration. Error bars-SEM. *P* values by students unpaired *T* test: *P < 0.05

Finally, we examined whether the RARa agonist may protect the brain from LPS-induced neurodegeneration. Given the literature on the early breakdown of the blood–brain barrier in AD patients and AD mice (42) and evidence on increased LPS levels in the AD brain (10,11,43), we performed further experiments by direct ICV delivery of LPS (Fig. 5A). This approach enabled to minimize any systemic effects, and specifically any systemic immune responses to LPS, as well as to examine whether AD pathology underlies the brains’ vulnerability to LPS neurotoxicity. First, a single 25 µg LPS dose was injected ICV into 7-month-old wt mice, in which Am580 (or vehicle) was delivered continuously via mini-osmotic pumps. LPS injection caused a significant loss in cortical neurons (approximately 25% loss from age-matched wt mice), which was partially prevented by Am580 (Fig. 5B–D). ICV injection of a similar dose of LPS into 5xFAD mice caused profound neuronal loss (approximately 50% loss), which was not affected by Am580 (Fig. 5E–G). We therefore injected a low dose (10 µg) of LPS into 5xFAD mice brain ventricles. This caused mild loss of cortical neurons (approximately 25%), which was partially prevented by the Am580 treatment (Fig. 5H–J). To confirm that the neuroprotective effect of the RARα agonist was not restricted to TLR4-mediated neurotoxicity of LPS, we injected 25 µg Zymosan ICV to wt mice in which Am580 (or vehicle) was delivered continuously via mini-osmotic pumps. Am580 provided efficient protection against Zymosan-induced cortical neuronal loss (Fig. 5K–M), supporting the notion that microglial modulation may protect from PAMP-induced neurodegeneration.

Thus, ICV injection of LPS induces a dose-dependent loss of cortical neurons in 5xFAD mice, and the amyloid-burdened brain is more susceptible to LPS neurotoxicity than wt brains. Furthermore, continuous ICV delivery of Am580 partially protects from LPS-induced neurodegeneration. These support the notions that repeated exposures to LPS may result in cumulative neurodegeneration, and that brains inflicted with AD pathology show increased vulnerability to infectious agent-derived PAMP neurotoxicity. Furthermore, these data indicate that PAMP-induced neurotoxicity is indeed mediated at least in part by microglia and is reversible by microglial modulation.

**Fig. 6:**
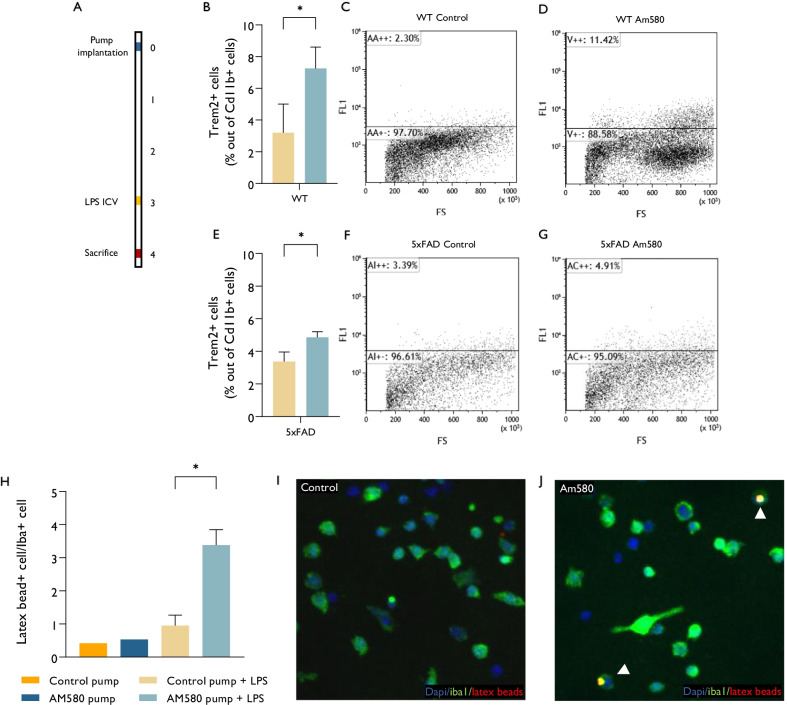
Am580 restores microglial homeostatic phenotype. **A** 5xFAD mice were implanted with pumps containing either Am580 or vehicle. 3 days later, mice were injected with LPS ICV. Mice were sacrificed on the following day for isolation of brain microglia, which were either stained for FACS analysis (**B**–**G**) or cultured with LPS for 1 h for latex beads phagocytosis quantification (**H**–**J**). Am580 caused an increase in the fraction of neuroprotective (TREM2+) microglia in either wt and 5xFAD mice (**B**–**G**, *n* = 3). Am580 induced an increase in microglial phagocytic activity. **H** Quantification of microglia that phagocytized latex beads out of total iba1 + cells. **I**, **J** Representative images of the Iba1 + microglia and phagocyted latex beads. **I** Microglia isolated from Vehicle treated mice, **J** microglia isolated from Am580 treated mice

To further characterize the modulatory effect of Am580 on microglial response to LPS in vivo, we isolated CD11b + microglia from Am580- or vehicle-treated mice after LPS injection and assessed their function ex vivo (Fig. 6A). In both wt (Fig. 6B–D) and 5xFAD (Fig. 6E–G)—Am580-treated mice there was an increase in the fraction of (neuroprotective) TREM2 + microglia. TREM2 is involved in the phagocytic functions of microglia (44,45). In agreement, Microglia from LPS-injected, Am580-treated brains exhibited increased ability to phagocytose latex beads (Fig. 6H–J). Am580 did not affect significantly the overall expression of TLR2 and TLR4 (data not shown), suggesting that its protective effect was not mediated by simple downregulation of the LPS receptor, TLR4.

Thus, Am580 does not suppress microglial activity, but rather shifts them towards a homeostatic and neuroprotective phenotype and reduces their LPS-induced neurotoxicity.

In conclusion, AD mice exhibit increased vulnerability to endotoxin-induced neurotoxicity, a process mediated by activated microglia. Delivery of a RARα agonist into brains with AD pathology modulates the neurotoxic phenotype of microglia and protects the brain from PAMP-induced neurodegeneration.

## Discussion

We show here that exposure to subclinical systemic infections by housing in a natural environment is associated with accelerated neurodegeneration in AD mice. Brain susceptibility to systemic infections was unique to mice with AD brain pathology, as housing of wt mice in “dirty” conditions had no deleterious effects on cortical neurons. The accelerated neurodegeneration in AD mice occurred as a direct effect of the environmental exposure, without inducing a significant change in the principal pathological hallmarks of AD, namely, Aβ deposition and microgliosis. Accelerated neurodegeneration occurred in the microglia-rich frontal cortex, but not in the microglia-poor CA1 and CA3 regions of the hippocampus. As LPS endotoxin is a major PAMP derived from infectious agents that are excluded from the SPF facility, we modeled environmental exposure to infectious agents by injecting LPS systemically. LPS caused significant loss of cortical neurons in AD, but not wt mice. Direct ICV delivery of LPS induced a dose-dependent loss in cortical neurons, with significantly higher susceptibility of AD mice to its neurotoxicity. Finally, regulation of microglial activation by a retinoic acid receptor α agonist prevented LPS-induced neurodegeneration. The RARα agonist reduced also microbial TLR2 agonist-induced cortical neuronal loss, confirming the concept that microglial modulation may protect from PAMP-induced neurodegeneration.

Our study on the effect of systemic pathogens on neurodegeneration in AD may be clinically relevant, as it supports the notion that systemic infections are associated with cognitive worsening and neurodegeneration in AD (46–49). First, our findings suggest that acute systemic bacterial infections may not only affect cognitive functions by inflammation-induced sickness behavior but also induce the irreversible loss of cortical neurons. This is supported by a recent study showing that even a single systemic LPS injection induced an increase in pathological changes in a mouse model of human Tauopathy, associated with a long-term decline in behavioral and motor function (50). Second, our study suggests that chronic asymptomatic colonization with endotoxin-producing pathogens and recurrent subclinical infections may accelerate neurodegeneration in AD as well. This is supported by our finding which housing 5xFAD mice in non-SPF conditions induced neurodegeneration, without clinically apparent infections, and that systemic delivery of LPS at a low dose (that did not cause clinical manifestations) induced neurodegeneration as well. Indeed, a large body of evidence suggests bidirectional relationships of the gut–brain axis (51,52). AD related changes in the enteric nervous system may affect the gut microbiome (53), and reciprocally altered gut microbiome may facilitate AD pathogenesis in the brain through its related neuro-hormones, PAMPs and microbial-derived amyloidogenic proteins (51,52,54). In agreement, recent studies showed that long-term broad-spectrum combinatorial antibiotic treatment decreases AD pathology (55) and that germ-free and antibiotic-treated 5xFAD mice exhibited reduced hippocampal AD pathology and neuronal loss (56).

The notion that LPS injection can model the effect of systemic pathogens on neurodegeneration in AD is supported by several observations. It has been shown that blood LPS levels are higher in AD patients as compared to age-matched cognitively intact patients (10,11) and that LPS is found in AD amyloid plaques (11,12). Gram-negative bacteria, containing endotoxin, are found at a very high volume in the mammalian gut, skin, and oral mucosa. The human body carries approximately 1 g of endotoxin, whereas systemic administration of just 100 ng may induce strong inflammation in the body and brain (57). Injection of LPS systemically causes marked microglial activation directly (58), and independently of the presence of its TLR4 receptor on peripheral immune cells (59). Furthermore, activated microglia have been shown to mediate neuronal death in AD mice (25,27,60). It has been controversial whether LPS penetrates the intact brain or affects the brain indirectly (57). Activation of microglia by systemic administration of LPS may be mediated indirectly via systemic–CNS interfaces, such as the blood–brain barrier, choroid plexus, and activation of peripheral nerves acting centrally and of circumventricular organs. However, recent evidence showed that the breakdown of the blood–brain barrier in AD patients and AD mice occurs very early (42), enabling increased penetration of LPS to the CNS (61). In support of the endotoxin theory of AD, suggesting that endotoxins from systemic microbial gram-negative bacteria play a role in the pathogenesis of AD (57), we provide evidence that microbial endotoxin directly accelerates neurodegeneration, and that this occurs depending on pre-existing AD pathology in the brain. The AD pathology, consisting of deposits of Aβ and microgliosis induced the brain’s susceptibility to the neurotoxic effects of the microbial-PAMP. In the 5xFAD mouse, the AD pathology encompasses the entire cortex. However, in the human brain, where AD pathology may start focally, the neurotoxicity of infectious agents may come into effect in a non-diffuse manner and induce neuronal death only in AD pathology-rich areas. This makes it difficult to differentiate pathologically between the basic misfolded protein-driven neurodegenerative process and PAMP-induced neuronal death, which is masked. Our finding that neurodegeneration in 5xFAD mice housed in natural environment is absent in the CA regions of the hippocampus, in which microglial density is low, strongly supports the hypothesis that AD pathology itself does not lead directly to neurodegeneration, rather induces microglial susceptibility to neurotoxic activations.

There is increasing understanding of the role of microglia in mediating neurotoxicity and neuronal death in AD. Our study suggests that microglia may mediate neuronal death not only in response to endogenous misfolded proteins, but also in response to exogenous insults, and in particular to systemic PAMP. Importantly, there are several populations of microglia in the AD brain, with different specializations, in which some are neurotoxic and others are neuroprotective (62,63). Furthermore, there is significant traffic of immune cells between the CNS and the systemic circulation that have an important role in AD pathogenesis (64–67). Here we did not distinguish between the various microglial phenotypes, infiltrating monocytes and CNS-associated macrophages. It should be noted that microbial PAMPs induce activation of other brain cells as well. Finally, astrocytes are also key players in AD pathogenesis, and respond to endotoxin by neurotoxic activation, may possibly contribute to the phenomenon of PAMP-induced neurodegeneration (68–71). We show that the regulation of microglia can protect the brain from PAMP-induced neurodegeneration, underscoring microglia as a therapeutic target in AD. We emphasize the need to regulate microglial neurotoxic activation, without inhibiting their basic immune and physiological functions. Especially, microglial phagocytic activity is essential for the removal of Aβ and preventing the further progression of AD pathology. In this light, the use of RARα agonists is attractive, regulating the neurotoxic activation of microglia (as evident by iNOS expression), without silencing their basic immune functions (as evident by their cytokine response) and supporting their phagocytic activity. Retinoids have been previously shown to attenuate neuroinflammation (40), improve phagocytic function, and reduce neuropathology in transgenic mouse models of AD (41,72–74). In agreement, we show that Am580 treatment increased the fraction of TREM2 + microglia, and microglia from Am580-treated brains exhibited an increased ability to phagocytose latex beads. This indicates that shifting microglial activity towards a neuroprotective phenotype using Am580, rather than suppressing microglia entirely, may be of therapeutic value in AD. In the future, targeted modulation of specific populations of brain immune cells may prove advantages over the non-cell type specific RARα activation that was performed here.

## Conclusions

AD mice exhibit increased vulnerability to an infectious environment and to endotoxin-induced neurodegeneration, a process mediated by activated microglia. Delivery of a RARα agonist into brains with AD pathology modulates the neurotoxic phenotype of microglia and protects the brain from PAMP-induced neurodegeneration.

## Data Availability

No supporting data besides presented in the manuscript.

## Abbreviations

PAMP: Pathogen associated molecular patterns
AD: Alzheimer’s disease
TLR: Toll-like receptor
SPF: Specific pathogen free
Aβ: Amyloid beta
PRRs: Pattern recognition receptors
ROS: Reactive oxygen species
iNOS: Inducible nitric oxide synthase
WT: Wild type
RARα: Retinoic acid receptor alpha
ICV: Intracerebroventricular
ANOVA: Analysis of variance
